# Evaluation of Phytase Producing Bacteria for Their Plant Growth Promoting Activities

**DOI:** 10.1155/2014/426483

**Published:** 2014-01-23

**Authors:** Prashant Singh, Vinod Kumar, Sanjeev Agrawal

**Affiliations:** ^1^Department of Biochemistry, College of Basic Sciences and Humanities, G. B. Pant University of Agriculture and Technology, Pantnagar 263145, India; ^2^Akal School of Biotechnology, Eternal University, Baru Sahib, Sirmaur 173101, India

## Abstract

Bacterial inoculants are known to possess plant growth promoting abilities and have potential as liquid biofertilizer application. Four phytase producing bacterial isolates (phytase activity in the range of 0.076–0.174 U/mL), identified as *Advenella* species (PB-05, PB-06, and PB-10) and *Cellulosimicrobium* sp. PB-09, were analyzed for their plant growth promoting activities like siderophore production, IAA production, HCN production, ammonia production, phosphate solubilization, and antifungal activity. All isolates were positive for the above characteristics except for HCN production. The solubilization index for phosphorus on Pikovskaya agar plates was in the range of 2–4. Significant amount of IAA (7.19 to 35.03 **μ**g/mL) production and solubilized phosphate (189.53 to 746.84 **μ**g/mL) was noticed by these isolates at different time intervals. Besides that, a greenhouse study was also conducted with Indian mustard to evaluate the potential of these isolates to promote plant growth. Effect of seed bacterization on various plant growth parameters and P uptake by plant were used as indicators. The plant growth promoting ability of bacterial isolates in pot experiments was correlated to IAA production, phosphate solubilization, and other *in vitro* tests. On the basis of present findings, isolate PB-06 was most promising in plant growth promotion with multiple growth promoting characteristics.

## 1. Introduction

It is well known that a considerable number of bacterial species, mostly those associated with the plant rhizosphere, are able to exert a beneficial effect upon plant growth. Therefore, their use as biofertilizers or control agents for agriculture improvement has been a focus of numerous researchers for a number of years. Bacterial inoculants have been used to increase plant yields in several countries, and commercial products are currently available. For example, in India, several biofertilizers are commercially produced and employed with different crops, mostly using strains of *Azotobacter, Rhizobium, Azospirillum*, and *Burkholderia*. Several possible mechanisms have been proposed, including suppression of diseases caused by plant pathogens [1], competition with pathogenic microorganisms by colonizing roots [2], production of plant-growth-regulating substances such as indole-3-acetic acid (IAA) [3], and lowering ethylene levels in root cells. Plant-stimulatory effects exerted by plant growth promoting bacteria (PGPB) might also be due to an enhanced availability of limited plant nutrients such as nitrogen, phosphorus, B-vitamins, and amino acids in the rhizosphere caused by phosphate-solubilizing and diazotrophic bacteria [4, 5]. Improved phosphorus nutrition is achievable by “mobilization” of phosphorus as insoluble inorganic polyphosphates and phytate, which accounts for 20–50% of the total soil organic phosphorus [6]. A number of PGPB are efficient in phytostimulation and biofertilization, and as biocontrol agents, but in most cases difficulties in obtaining successful formulations and insufficient knowledge of the basic molecular principles of their action has hindered their commercial use [7].

P is an important plant macronutrient, making up for about 0.2% of a plant's dry weight. It is a component of key molecules such as nucleic acids, phospholipids, and ATP, and, consequently, plants cannot grow without a reliable supply of this nutrient. P is also involved in controlling key enzyme reactions and in the regulation of metabolic pathways. After N, P is the second most frequently limiting macronutrient for plant growth. In the rhizosphere, the conversion of the insoluble forms of inorganic P to a form accessible by plants is achieved by phosphate-solubilizing bacteria (PSB) which release phosphates meanly by organic acids releasing. However, organic P forms, particularly phytates, are predominant in most soils (10–50% of total P) and must be mineralized by phytases (myo-inositol hexakisphosphate phosphohydrolases) to be available P for plants [8]. In this context, bacteria with both activities, production of organic acids to solubilise inorganic P and production of phytase to mineralize phytate, have been isolated from rhizosphere [9, 10] and proposed as potential plant growth promoting rhizobacteria (PGPR) to be used in soil with high content of organic P. Studies have also revealed that phytase-producing rhizobacteria (PPB) not only harbor the ability to mineralize phytate but also harbor other PGPR activities, such as the production of indole acetic acid, siderophore, volatiles, and ammonia [11, 12]. Indian mustard (*Brassica juncea*) is an important oil seed crop, which produces an edible mustard oil used in vegetables, pickles, and so forth, at a large scale. The present study was conducted with the following objectives of characterization of phosphate solubilizing bacterial isolates for plant growth promoting potential *in vitro* and a greenhouse study for the ability of selected isolates to promote plant growth of mustard (*Brassica juncea*). Recently, a similar study was conducted with mustard using *Achromobacter *sp., *Bacillus* sp., and *Tetrathiobactor* sp. [13].

## 2. Material and Method

### 2.1. Chemicals, Reagents, and Media

Microbiological media like Pikovskaya agar, Luria Broth (LB) media and nutrient agar media were procured from HiMedia Pvt. Ltd., India. Other chemicals of interests used were of analytical grade. Previously isolated phytase producing bacterial strains (PB-05, PB-06, PB-09, and PB-10) in our lab was taken for the following study [14].

### 2.2. Production and Determination of Phytase Activity

The isolates were tested for their ability to produce phytase on the phytase screening medium (PSM) described by Kerovuo et al. [15]. The streaked plates were incubated overnight at 37°C and the translucent region of the plate gave a visual indication of extracellular phytase production. Phytase activity in liquid PSM media was determined by following the method of Engelen et al. [16] as used in Kumar et al. [13].

### 2.3. Analysis of Phosphate Solubilization Efficiency

Phosphate solubilization by bacterial isolates was done by the method of Pikovskaya [17]. Plates were made in duplicate for each bacterial isolate using Pikovskaya agar medium. Bacterial culture was point inoculated at the centre of Pikovskaya agar plate and incubated in incubator at 28°C for 7 d. The plates were then examined for halo zone around bacterial culture and solubilization index (S.I.) was calculated as: S.I. = (colony diameter + halo zone diameter)/colony diameter [18].

Phosphate solubilizing efficiency of bacterial cultures was quantified in specified NBRIP (national botanical research institute phosphate: glucose 10 g/L, Ca_3_(PO_4_)_2_ 4 g/L, MgCl_2_·6H_2_O 5 g/L, MgSO_4_·7H_2_O 0.25 g/L, KCl 0.2 g/L and NH_4_(SO_4_)_2_ 0.1 g/L) media using the method of Nautiyal [4].

### 2.4. Screening for Other Plant Growth Promoting Activities

#### 2.4.1. Indole Acetic Acid, Siderophore, Hydrogen Cyanide, Organic Acid, and Ammonia Production

Indole acetic acid (IAA) production by bacterial isolates was determined in LB broth supplemented with L-Tryptophan (500 *μ*g/mL) at 24, 48, and 72 h as described by Patten and Glick [19]. For this, bacterial cells were removed by centrifugation at 10,000 rpm for 5 min at 4°C. One mL of the supernatant was mixed with 4 mL of Salkowski's reagent in the ratio of 1 : 4 and incubated at room temperature for 20 min. Development of a pink colour indicated indoles. The absorbance of supernatant mixture (supernatant + Salkowski's reagent) for indole production was measured at 530 nm and quantity of indoles was determined by comparison with a standard curve using an IAA standard graph. Siderophore production was determined by using blue indicator dye and chrome azurol S agar [20]. Bacterial isolates exhibiting orange halo zone on chrome azurol S agar after 5 d of incubation at 28°C were considered positive for the production of siderophores. For hydrogen cyanide (HCN) production the methodology described by Bakker and Schippers [21] was used. Isolates were grown on plates of tryptic soy agar (10%), amended with glycine (4.4 g L^−1^), and FeCl_3_·H_2_O (0.3 mM). A change from yellow to orange, red, brown, or reddish brown was recorded as an indication of weak, moderate, or strongly cyanogenic potential, respectively. Organic acid production potential of different isolates was analyzed using thin layer chromatography (TLC) on Silica-G (Merck chemicals) gel plates using different solvent systems. Finally, ammonia production test was performed by growing selected isolated in peptone water for 72 h at 30°C. Change in colour after addition of 1 mL Nessler's reagent (K_2_HgI_4_; 1.4%) in each tube was observed. The presence of faint yellow colour indicates small amount of ammonia and deep yellow to brownish colour indicates maximum ammonia production.

#### 2.4.2. Antifungal Activity

Antagonistic nature of bacterial isolate against *A. brassicaceae* was determined by employing dual culture technique. Briefly, bacterial isolates were streaked at the centre of 90 mm petriplate containing PDA and incubated for 36 h at 25 ± 2°C. A plug of fungus was then placed on the edge of petriplate at both sides of streaked culture. Plates were incubated at 25 ± 2°C for 7 d. The radii of the fungal colony towards and away from the bacterial colony were measured. The percentage of growth inhibition was calculated using the formula % inhibition = (*R* − *r*)/*R*, where *r* is the radius of the fungal colony opposite the bacterial colony and *R* is the maximum radius of the fungal colony away from the bacterial colony [22].

### 2.5. Greenhouse Experiment

#### 2.5.1. Seeds and Soil

Seeds of *Brassica juncea* were surface sterilized with 1% sodium hypochlorite for 30 sec and rinsed with double distilled water thrice before use in greenhouse experiment. Soil used in the experiment was collected from the experimental farm of the university; the soil was dried and sieved to 2 mm before mixing it with cow dung manure (3 : 1) and autoclaved for 1 h prior to pot experiment. The chemical characteristics of soil mixture were also analyzed; used soil mixture contained 235.2 kg ha^1^ potassium, 200.7 kg ha^−1^ nitrogen, 28.22 kg ha^−1^ phosphorus, 1.5% organic acid content, 0.171 milisimon electrical conductivity, and 6.74 pH and the texture of soil was silt clay loam.

#### 2.5.2. Seed Bacterization and Pot Experiment

Bacterial cells were inoculated in 25 mL LB in 100 mL conical flask and incubated at 37°C, 120 rpm. Colony forming units (cfu) were counted and bacterial inoculums containing approximately 1 × 10^8^ cfu/mL were used for seed bacterization. CMC (100 mg) was added to flask containing culture inoculum as adhesive material. Ten gram of seeds was soaked in bacterial suspension for 12 h on a rotary shaker at 150 rpm. The bacterial suspension was drained off and the seeds were dried overnight aseptically in laminar air flow. Seeds soaked in distilled water amended with CMC served as control. The plants were grown in greenhouse under a day/night cycle of 16/8 h, 25/20°C, and 60% relative humidity. Pot soil (soil: decomposed cow dung manure at 3 : 1 v/v) was filled into pots (20 cm diameter). Ten bacterized seeds of *Brassica* were sown in each pot in three replicates.

#### 2.5.3. Phosphorus Content Estimation

30 d old seedlings were collected by uprooting the plants carefully without damaging the root system and analyzed for phosphorus content. Seedlings were air dried, grounded, and digested in 15 mL HClO_4_ and 5 mL HNO_3_ and phosphorus content was measured using spectrophotometric vanado-molybdate method [23]. Rhizosphere soil samples were collected from each treatment and analyzed for variation in pH and available phosphorous content by following Olsen's method [24].

### 2.6. Characterization of Bacterial Isolates

Bacterial isolates were taken from the culture during log phase for gram staining and were examined for endospore formation during stationary phase. Genetic characterization based on 16S rRNA gene sequence was also done. Briefly, genomic DNA from selected isolates was extracted as described by Neumann et al. [25], and PCR amplification of 16S rRNA gene was carried out by using primers: RDNA-1A (5′-AGA GTT TGA TCC TGG CTC AG-3′) and RDNA-1B (5′-AAG GAG GTG ATC CAG CCG CA-3′). The PCR was done as follows: a hot-start of 94°C for 3 min followed by 35 cycles of 94°C for 1 min, 54°C for 1 min, 72°C for 1.5 min, and a final extension for 10 min at 72°C. Amplified PCR products were purified with QIAquick Gel Extraction kit (Qiagen, Germany) and sequenced in an automated DNA sequencer (Applied Biosystems 3730) at DNA Sequencing Facility, University of Delhi (South Campus), New Delhi, India. The sequences obtained were compared with sequences in the NCBI GenBank database using blastn program (http://blast.ncbi.nlm.nih.gov/Blast.cgi) and then deposited in NCBI.

## 3. Result and Discussion

### 3.1. Characterization of Bacterial Isolates

All studied bacterial cultures were gram negative. Out of four bacterial isolates PB-05 were found to be endospore forming and showed green colour under microscope while the remaining bacterial isolates, that is, PB-06, PB-09, and PB-10 were nonendospore forming (Table 1). Based on 16S rDNA gene sequences, three isolates (PB-05, PB-06, PB-10) were identified as *Advenella *species (GenBank accession number JN630808.1, JN630809.1, and JQ727433.1 for PB-05, PB-06, and PB-10, resp.) while the fourth isolate was identified as *Cellulosimicrobium* sp. PB-09 (GenBank accession number JN630806.1). In another study by Kumar et al. [13], the isolates used for similar study were identified as *Achromobactor *sp. PB-01, *Bacillus* sp. PB-13, and *Tetrathiobactor* sp. PB-03.

### 3.2. Phytase Production and Phosphate Solubilization by Bacterial Isolates

In the soil, 20–80% of phosphate is in organic form [8] and plant may poorly/not possess an innate ability to acquire phosphorus directly from soil phytate [26]. Hence these isolated PSB's were analyzed for their phytase activity which degrades the soil phytate to lower phosphate esters which are available to plants. All of the four bacterial cultures showed translucent region around colonies on phytase screening medium described by Kerovuo et al. [15]. In liquid PSM media, all bacterial isolates were found to be positive for phytase production. Highest phytase activity was shown by PB-06 (0.174 U/mL) followed by PB-10 (0.161 U/mL), PB-09 (0.129 U/mL), and PB-05 (0.076 U/mL) (Table 2). Isolate PB-06 predominantly produced higher level of phytase. Nutrient source and concentration greatly influence the bacterial growth and enzyme production. High phytate content in the medium might induce the synthesis of phytase enzyme where 5 mM concentration of sodium phytate induced the phytase production. Phytase has been isolated and characterized earlier from several gram-positive and gram negative soil bacteria, for example, *Bacillus subtilis* [15], Acetobactor sp. [13], *B. laevolacticus* [27], *Klebsiella terrigena* [28], *Pseudomonas *sp. [29], and *Enterobacter* sp. [30]. Idriss et al. [31] reported that the extracellular phytase from *B. amyloliquefaciens *FZB45 promotes growth of maize seedlings under *in vitro* conditions. In another study, Kumar et al. [13] also reported phytase producing bacterial isolates promoting plant growth of Indian mustard.

**Figure 1:**
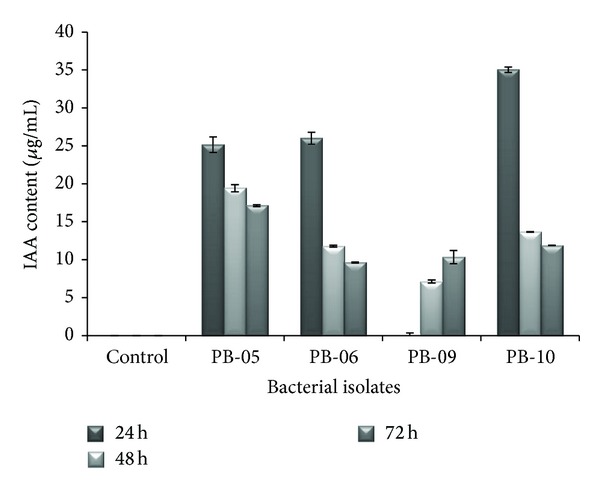
Effect of time on phosphate solubilization by bacterial isolates.

The bacterial isolates solubilized tricalcium phosphate in Pikovskaya media. *Advenella* sp. PB-06 showed highest solubilization index of 4, while *Cellulosimicrobium *sp. PB-09 and *Advenella* sp. PB-10 showed the least solubilization index of 2. *Advenella* sp. PB-05 showed the intermediate solubilization index of 2.5 (Table 1). Since the direct measurement of P solubilization in broth assay is likely to give more reliable results than regular plate assay, isolates were also tested for their ability to solubilize tricalcium phosphate (TCP) in NBRIP medium at 24 h, 48 h, and 72 h and were found to solubilize tricalcium phosphate with an increase from 24 h to 72 h in case of PB-09 while for other isolates it increases upto 48 h then it remains constant. Maximum phosphate solubilization shown by PB-09 at 72 h was 746.84 *μ*g/mL. Least phosphate solubilization shown by PB-05 at 24 h was 189.53 *μ*g/mL (Figure 1). A direct correlation of P-solubilizing activity in solid and liquid media was found in all tested isolates. They showed better P solubilizing activity in liquid medium compared to solid medium. On plates, the isolates P-solubilizing zone around the bacterial colony varied from 0.8 to 2.0 cm in radius while in liquid medium it varied from 189.53 to 746.84 *μ*g mL^−1^ using TCP as a source of insoluble P. The final pH of the medium in which this strain was grown increased from 7 to 8.74 after incubation. Similar studies have been conducted by Alikhani et al. [32], where different isolates of rhizobia from Iranian soils were tested for their ability to dissolve inorganic and organic phosphates. In contrast, they observed the drop in pH of the culture filtrate with the release of soluble orthophosphate which indicated the importance of organic acid production in the mobilization process. In a similar study, Kumar et al. [13] have also studied three phytase producing isolates for their P solubilization abilities on solid medium as well as liquid NBRIP media. In that case, solubilization index and P solubilized were in the range of 1.29–2.0 and 193–642 *μ*g mL^−1^, respectively.

### 3.3. Plant Growth Promoting Activities of Bacterial Isolates

PGPB can exert a direct effect on plant growth other than the mechanism of phosphate solubilization like production of phytohormones, biological nitrogen fixation, enhancing the availability of other trace elements, and increased iron nutrition through iron-chelating siderophores and volatile compounds that affects the plant signaling pathways. Additionally, by antibiosis, competition for space and nutrients and induction of systemic resistance in plant against a broad spectrum of root and foliar pathogens might also contribute to enhanced plant growth and metabolism [33]. Characterization of different traits of rhizobacteria was the common procedure employed while screening and selecting PGPB's [13, 34]. In our study too, these phytase producing bacterial isolates were characterized for different traits like IAA production, siderophore, HCN, antibiosis, and their plant growth promoting ability.

**Figure 2:**
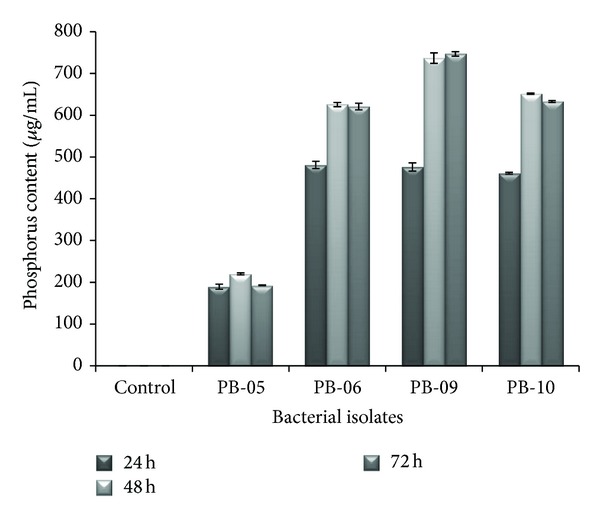
Effect of time on IAA production by bacterial isolates.

All bacterial cultures were IAA producing as indicated by observed pink colour in flasks of all cultures (except PB-09 at 24 h and control). At 48 h and 72 h, *Cellulosimicrobium *sp. PB-09 also produced IAA as indicated by pink colour of culture. IAA level in media increased from 24 h to 72 h in case of *Cellulosimicrobium* sp. PB-09, while in *Advenella *species, that is, PB-05, PB-06, and PB-10, it decreases from 24 h to 72 h. Maximum IAA production was shown by *Advenella *sp. PB-10 (35.03 *μ*g/mL) followed by *Advenella *sp. PB-06 (26.0 *μ*g/mL) at 24 h, while minimum was shown by *Cellulosimicrobium* sp. PB-09 (7.19 *μ*g/mL) at 48 h followed by *Advenella *sp. PB-06 (9.62 *μ*g/mL) at 72 h (Figure 2). These isolates released greater quantities of IAA in the presence of a physiological precursor, tryptophan in culture medium. Production of IAA varies greatly among different species and is also influenced by culture conditions, growth stage, and availability of substrate [35]. Leinhos and Vacek [36] reported IAA production by *Pseudomonas *and *Acinetobacter *isolated from wheat and rye rhizosphere ranging from 0.01 to 3.98 mg mL^−1^. Bacterial isolates obtained from the rhizosphere of various plants have been shown to produce IAA in pure culture. Most of the isolates in this study produced higher IAA in the presence of the precursor, L-Trp.

All the selected isolates were found positive for siderophores and ammonia production but negative for HCN production (Table 1). In our studies none of the isolates has shown HCN production (shows antifungal activity), which might be due to variation in growth parameters like temperature, nutrient availability, and growth pattern. All isolates showed NH_3_ production at a high extent due to N_2_ availability which increased to plant roots and plant could synthesize nitrate for uptake. When bacterial isolates on PDA plate were challenge inoculated with pathogenic fungi like *Alternaria brassicacae,* isolates PB-06 and PB-10 afforded some degree of inhibition against fungal growth and showed antagonism against *A. brassicaceae *under *in vitro* experiments. Bacterial isolates PB-06 and PB-10 showed 18.75% and 25% fungal (*A. brassicacae*) growth inhibition, respectively. Bacterial isolates PB-05 and PB-09 showed no antifungal activity in dual culture technique (Table 2). The protection offered by the isolates may be due to induced systemic resistance. But in other isolates no fungal growth inhibition observed provided resistance over the isolates.

### 3.4. Greenhouse Studies

Phosphorus solubilization is one of the important mechanisms through which PGPB isolates promote plant growth, but this is not the only way of plant growth promotion. There are several other mechanisms like direct stimulation, production of gibberellins, cytokinin, ACC deaminase, and volatile compounds that are also reported previously [37], which were not characterized in the present study. *Brassica *seeds bacterized with rhizospheric isolates showed significant increased shoot length, root length, fresh weight, and dry weight over control. Also, all bacterial isolates inoculated plants showed an increased inorganic P content in seedlings. *Advenella *sp. PB-06 inoculated plant showed highest increase in root length (6.38 cm) and *Advenella *sp. PB-10 inoculated plants showed highest increase in shoot length (14.30 cm) (Table 2). It was assumed that one or many of these traits may be involved in the plant growth promoting activity by isolates PB-05, PB-06, PB-09, and PB-10. Our result showed the successful screening of the bacterial isolates for *in vitro* solubilization of inorganic phosphate, IAA production, and their effects on fresh weight, dry weight, root length, and shoot length of *Brassica *seedlings. Seedlings containing *Cellulosimicrobium *sp. PB-09 showed highest P content of 302 mg/g. Maximum available P in soil enhanced by *Advenella *sp. PB-06 was 8.77 mg/Kg soil. Seedlings raised from seeds bacterized with phytase positive PGPB isolates showed higher accumulation of phosphorus content than uninoculated control. Among them isolate PB-09 treatment showed significant increase in accumulation of phosphorous in comparison with uninoculated. Isolate PB-09 showed significant increase in phosphorus accumulation in seedlings while isolate PB-05 was found significantly increasing dry weight of seedlings in comparison to control (Table 2). Several rhizobacteria can solubilize inorganic phosphate from soil and such bacteria improve solubilization of unavailable soil phosphate resulting in a high efficiency of phosphorus use. When rhizosphere soil samples of 30 d old seedlings, with all PSRB isolates, were analyzed, not much variations in pH was observed. There were no significant changes in pH of soil samples collected from different seedlings.

In conclusion, All studied phytase-producing bacteria from Himalayan soils showed ability to harbor diverse plant growth promoting activities, including production of phytase, ammonia, siderophores and indole acetic acid, releasing of P from insoluble inorganic phosphates, and inhibition of phytopathogen *R. solani*. Also, the inoculation of *Brassica juncea* seeds with *Advenella *species (PB-05, PB-06, and PB-10) showed improved P content and growth of *Brassica juncea*. The present study clearly revealed that all isolates tested in this study had the ability to solubilize inorganic phosphate, producing phytase, ammonia, and IAA and increased availability of P, IAA, and ammonia leading to increased plant growth. These isolates may be further characterized using molecular approaches and changes in expression of related genes for in-depth understanding of detailed mechanism of plant growth promotion. The isolates may also be used in development of a suitable liquid biofertilizer, employing a consortium of such kind of plant beneficial microbes.

## Figures and Tables

**Table 1:** Plant growth promoting characters of phytase producing bacterial isolates.

Isolates	Characterization (staining)		Production assay				Phosphorus solubilization		
	Gram's	Endospore	Siderophore	Ammonia	HCN	Catalase	Ca_3_(PO_4_)_2_	Phytate	Solubilization index (Ca_3_(PO_4_)_2_)
Control	NA	NA	−	−	−	−	−	−	0
PB-05	−	+	+	++	−	+	+	+	2
PB-06	−	−	+	++	−	+	+++	+	4
PB-09	−	−	++	+	−	+	+	+	2
PB-10	−	−	+	++	−	+	++	+	2.5

**Table 2:** Effect of seed bacterization on different plant growth parameters after 30 days of germination under greenhouse study.

Bacterial isolates	Fresh weight	Dry weight	Root length	Shoot length	P content	Antifungal activity	Phytase activity
	(g)	(g)	(cm)	(cm)	(mg/g)	% inhibition	(U/mL)
Control	0.182 ± 0.003	0.039 ± 0.002	6.740 ± 0.050	3.540 ± 0.029	143.0 ± 2.04	0.00	0.000
PB-05	1.380 ± 0.029	0.455 ± 0.001	13.340 ± 0.136	5.720 ± 0.091	227.0 ± 3.00	0.00	0.076
PB-06	1.030 ± 0.018	0.160 ± 0.005	13.380 ± 0.115	6.380 ± 0.152	220.5 ± 9.59	18.75	0.174
PB-09	0.629 ± 0.002	0.082 ± 0.003	10.620 ± 0.096	4.200 ± 0.118	302.0 ± 4.07	0.00	0.129
PB-10	1.016 ± 0.027	0.132 ± 0.007	14.300 ± 0.114	4.460 ± 0.067	178.0 ± 6.01	25.00	0.161
